# Syndemic factors associated with adherence to antiretroviral therapy among HIV-positive adult heterosexual men

**DOI:** 10.1186/s12981-019-0248-9

**Published:** 2019-11-09

**Authors:** James M. McMahon, Amy Braksmajer, Chen Zhang, Natalie Leblanc, Michael Chen, Angela Aidala, Janie Simmons

**Affiliations:** 1 School of Nursing, University of Rochester Medical Center, 601 Elmwood Avenue, Box SON, Rochester, NY 14624 USA; 2 Department of Public Health Sciences, School of Medicine and Dentistry, University of Rochester Medical Center, Rochester, NY USA; 3 Mailman School of Public Health, Columbia University, New York, NY USA; 4 National Development and Research Institutes, New York, NY USA

**Keywords:** Antiretroviral therapy, Adherence, Viral suppression, Stigma, Social support, Heterosexual men, HIV-positive, Structural equation modeling

## Abstract

**Background:**

Suboptimal adherence to HIV antiretroviral therapy (ART) and concomitant lack of viral control can have severe consequences for health and onward transmission among persons living with HIV. Little is known about the barriers and facilitators of optimal ART adherence among heterosexual HIV-positive men.

**Methods:**

Structural equation modeling (SEM) was performed to test a theory-derived model of ART adherence using data from a cross-sectional sample of 317 HIV-positive self-identified heterosexual men residing in New York City. We assessed a conceptual model in which mental health (depression, anxiety) and substance use dependence mediated the effects of socio-structural factors (HIV-related stigma, social support) on ART adherence, and subsequently, undetectable viral load.

**Results:**

Structural equation modeling analyses indicated that men who reported higher levels of HIV-related stigma tended to experience higher levels of general anxiety, which in turn was associated with reduced probability of optimal ART adherence. Moreover, men who reported higher levels of social support tended to exhibit less dependence on illicit substance use, which in turn was associated with increased probability of optimal ART adherence. African-American men reported lower ART adherence compared to other racial/ethnic groups.

**Conclusions:**

Our findings support the hypothesis that substance use dependence and mental health problems, particularly anxiety, may be primary drivers of suboptimal ART adherence among heterosexual men, and that socio-structural factors such as HIV-related stigma and social support are potential modifiable antecedents of these drivers.

## Introduction

Optimal adherence to HIV antiretroviral therapy (ART) is essential for sustained HIV-1 RNA viral suppression. Suboptimal ART adherence can lead to lack of viral control and adverse clinical outcomes among people living with HIV/AIDS (PLWHA), including the emergence of drug-resistant virus [1–4], virologic failure [5–7], accelerated disease progression, [8–10], and increased risk of secondary HIV transmission [11, 12]. Recent evidence also indicates that suboptimal ART adherence (i.e., < 100%) can lead to increased residual inflammation, despite viral suppression [13]. A meta-analysis of studies examining ART adherence found that among 6777 ART patients in the U.S., only 69% were optimally adherent [14]; and more than 1 in 4 of those who engaged in HIV care lacked viral control [15]. Suboptimal ART adherence thus represents a prevalent and serious barrier to effective treatment.

While ART adherence has been well-studied in men who have sex with men [16–18], minority women [19, 20], and other groups [21], few studies have specifically examined the determinants of suboptimal ART adherence among HIV-positive men who have sex with women (MSW). Heterosexual men make up about 8% of people living with HIV (PLWH) nationally, and there were an estimated 2829 new HIV diagnoses among MSW in 2017 [22]. Analysis of 2014 data from the National HIV Surveillance System revealed that 46% of HIV-positive MSW failed to achieve viral suppression, and even among those engaged in HIV care 26% did not achieve viral suppression [23, 24]. This is noteworthy, given that HIV-positive MSW are mostly men of color (61% African American and 22% Latino) and increasingly older [22], factors that are associated with worse disease progression [25, 26]. Among people age 50 and older who received an HIV diagnosis in 2016, 15% were heterosexual men [27]. From 2005 to 2010, the proportion of HIV-positive MSW who survived > 36 months after diagnosis was 88%, compared to 95% for men who have sex with men (MSM) and 92% for women. Among HIV-positive MSW with a stage 3 AIDS diagnosis, 6732 deaths were reported from 2012 to 2016 [22]. In addition, HIV-positive MSW constitute the primary risk group for HIV transmission to women and girls: about 85% of the 6341 new infections among female adults and adolescents in 2017 were attributed to sexual contact with MSW [22]. Understanding the factors that promote or inhibit ART adherence among HIV-positive MSW can inform the development of targeted interventions leading to more effective treatment and prevention.

The processes affecting ART adherence are varied and complex, operating at multiple levels and diverse mechanisms. Use of a conceptual model is therefore essential for identifying relevant constructs, generating hypotheses, and providing a framework for interpretation of results [28, 29]. One of the most informative and predictive models of HIV-related outcomes is syndemic theory, which postulates that co-occurring adverse factors interact to contribute to excess disease burden or risk behaviors, including suboptimal ART adherence [30]. Syndemic factors related to adverse HIV outcomes often center on substance use disorders, mental health, and interpersonal factors (e.g., social support, intimate partner violence), and often cluster with social-structural factors that may attenuate or exacerbate HIV-related outcomes [31].

Across multiple studies, HIV-related stigma, social support, substance abuse, and mental health have been shown to be among the strongest predictors of HIV-related outcomes, including ART adherence [32, 33]. HIV-related stigma has been shown to compromise patients’ ability to successfully adhere to ART via affective, behavioral and physical pathways [34]. Anxiety, depression, and substance use disorders are known to impair psychological and cognitive processes [35–39], which could become manifest in lower levels of ART adherence [40–42]. For example, impairments in attention, working memory, decision-making, executive and global functioning, and psychomotor processing have all been linked to lower ART adherence [43–45]. In contrast, increased social support has been shown to play a critical role in maintaining ART adherence [46–48].

The current study evaluated a model on the effects of selected psychosocial factors (stigma and social support) on ART adherence, mediated by the syndemic constructs of severity of drug dependence and anxiety, among MSW living with HIV in New York City. We hypothesized that (1) higher levels of HIV-related stigma will be associated with higher depression, anxiety, and drug dependence, which, in turn, will be associated with lower levels of ART adherence; and (2) higher levels of social support will be associated with lower levels of depression, anxiety, and drug dependence, which will be associated with higher ART adherence. In addition to these indirect effects, we examined the association between self-reported ART adherence and viral suppression. Selected covariates were adjusted for in the model.

## Methods

Study data were drawn from the “Men’s Talk on HIV Risk” (MENTOR) Project, a cross-sectional retrospective survey of 317 heterosexual African American and Latino men with self-reported HIV/AIDS, recruited through health and social services agencies in Harlem and the South Bronx, NY, in 2011 and 2012.

### Target population and eligibility

The MENTOR Project focused on the health, sexual behavior, and services utilization of HIV positive men who have sex with women. Participants were eligible if they met the following criteria: (1) cis-gender male, (2) HIV-positive by self-report, (3) between 18 and 60 years of age, (4) conversant in English or Spanish, (5) self-identified as heterosexual, and (6) had vaginal or anal sex with a woman in the last 3 months. Individuals were excluded if they demonstrated cognitive impairment due to drug or alcohol use. Eligibility screening was determined via a brief phone interview prior to scheduling an interview for study enrollment.

### Sampling and recruitment

To access the target population, a two-stage sampling process was followed. In the first stage, 656 agencies were identified that provided services to people living with HIV/AIDS in Harlem and the South Bronx, NY. These agencies were extracted from the Community Health Advisory & Information Network (CHAIN) Project, a prospective study of representative samples of persons living with HIV/AIDS in New York City. The list of agencies was then randomly ordered, and agency directors were contacted and invited to participate in the project in the sequence in which they appeared on the random list. In an attempt to recruit younger HIV infected heterosexual men, agency sampling was stratified with the addition of a separate randomly ordered list of agencies serving younger (age 18–35 years) adults living with HIV. Contact was made or attempted with 121 randomly ordered agencies; 76 (63%) agreed to assist with study recruitment, and 45 (37%) declined participation or did not respond. In the second stage, informational flyers and cards were disseminated to providers to recruit participants using a criterion-based sampling process. Potential participants were only screened for eligibility if they reported receiving services from a collaborating agency. Potential participants who called a toll-free project recruitment number, were administered a brief eligibility screening, informed about the study, and, if eligible and interested, scheduled for an interview. At the project field office, eligible participants voluntarily signed an IRB-approved informed consent and were administered the quantitative structured survey. Each participant received 50 USD reimbursement.

### Data collection and measures

Structured quantitative interviews were administered in either English or Spanish by trained bilingual interviewers. Interviews lasted approximately 1.5 h, with sufficient breaks, and were conducted in a private office using computer-assisted personal interview (CAPI) software (QDS ver. 6.2.1, Nova Research).

#### Outcome measures

*Antiretroviral medication adherence* was measured by a single survey item on self-reported number of missed doses during the last month, dichotomized as no missed doses (100% adherence) versus any missed doses (< 100% adherence) [49], as less than 100% adherence has been linked to adverse biological effects in PWLH on ART [13, 50]. Although several studies have found that self-reported measures of adherence tend to over-estimate actual adherence levels [51], a recent meta-analysis showed that self-reported measures were equivalent to electronic monitoring, pill count, and pharmacy refill methods in the ability to predict virologic failure [52].

*Undetectable viral load* was a secondary outcome in the model. Participants were asked to report their viral load count from their most recent viral load test. Nearly all (98%) reported having received a viral load test within the last 6 months. We dichotomized this measure as viral load ≤ 50 copies/mL (undetectable viral load) versus > 50 copies/mL (detectable viral load). Sewell and colleagues assessed agreement between self-reported and clinic-recorded viral load among 2678 HIV patients and found that only 2.1% incorrectly self-reported their viral load as detectable when it was undetectable; but 22.1% incorrectly self-reported their viral load as undetectable when it was detectable, based on clinic records [53].

#### Predictor variables

*HIV*-*related stigma* was assessed using 8 items from the multidimensional HIV stigma scale, which included items measuring enacted, anticipated, and internalized HIV-related stigma [54]; response range: 1–5 (higher scores indicated greater stigma). Cronbach’s alpha, a measure of scale reliability, was .85. *Social support* was measured with the Lubben Social Network Scale (LSNS-6), which contains 3 items each on family and friend social network size [55]; response range: 0–5; Cronbach’s alpha: .82. *Depression* was measured using 7 items from the Client Diagnostic Questionnaire (CDQ) covering physiological symptoms, mood, and negative affect [56]; response range: 1–4; Cronbach’s alpha: .79. *Anxiety* was assessed using 6 items from the CDQ, which documented the frequency of men’s self-reported feelings of nervousness, anxiety, worry, restlessness, fatigue, sleep problems, lack of concentration, and irritability [56]; response range: 1–4; Cronbach’s alpha: .78. *Substance use dependence* was measured with the Severity of Dependence Scale (SDS), a 5-item scale concerned with the psychological components of drug dependence [57]; response range: 0–3; Cronbach’s alpha: .78. Several covariates that were bivariate predictors of ART adherence were also entered into the model, including African American/Black race, number of HIV-related symptoms, and a latent variable measuring socioeconomic status, composed of three indicators: average monthly income, total number of years of education, and whether employed.

### Statistical analysis

Standard data cleaning methods were applied to identify data errors and assess and remedy violations of analytical assumptions using SAS (ver. 9.2). Descriptive statistics were performed to characterize the sample and examine men’s self-reported reasons for missed ART doses. The analysis data set contained < 1% missing data overall, but produced 27 missing cases under listwise deletion. We therefore applied a full information maximum likelihood (FIML) approach to handling missing data under the assumption of MAR. All scale measures were treated as latent variables using structural equation models (SEM). Although numerous analytical approaches have been used to assess syndemic models [58], we employed SEM in order to explore relationships among the relevant predictors [59]. Bivariate logistic regression analyses were performed to estimate the effects of selected predictor variables on ART adherence, including demographics, HIV-related symptoms, mental and physical health indicators, substance use, and psychosocial and structural factors. Predictor variables identified to have a conclusive effect on the outcome (based on 95% CIs), were selected for SEM modeling based on syndemic theory. Diagnostic tests were performed to identify potential multicollinearity among the predictor variables. Structural equation modeling (SEM) was employed to test a syndemic model postulating direct and indirect relationships among variables. SEM models were performed using MPlus (ver. 8.1) with a weighted least squares with missing values (WLSMV) estimator and mixed linear and probit link functions, which can estimate models with combined continuous exogenous and dichotomous endogenous variables [60].

We followed recent guidelines set forth by the American Statistical Association not to employ the null hypothesis statistical testing framework and thus avoided the use of P-value cutoffs and wording related to the concept of “statistical significance” [61]; instead relying on interpretation of confidence intervals [62].

## Results

The study sample (N = 317) has been described in detail in prior publications [63, 64]. Briefly, the mean age was 47.7 years (SD = 6.6; range 20 to 59); mean number of years since HIV diagnosis was 15 (SD = 7.1); 64.5% were African American/Black and 29.7% were Hispanic/Latino; 35% had less than a high school education; 91.1% were unemployed, disabled or retired; 35.4% reported unstable housing; 60.6% had a primary female sexual partner (of whom, 53.7% were in an HIV-serodiscordant relationship with an HIV-negative partner); and 53.6% had used illicit drugs (marijuana, cocaine, crack, heroin, methamphetamines or hallucinogens) in the past 6 months.

Based on self-report, 94% (298/317) of participants were currently taking anti-retroviral medication, and 98% had a viral load test within the last 6 months. Among those on ART, 55.7% had achieved optimal ART adherence in the last month (see Table 1). Among all participants, 55.2% reported an undetectable viral load at their most recent VL test (56.0% among those taking ART). Optimal adherence was positively associated with an undetectable viral load (*r *= .39, *P *< .001; OR = 1.74, 95% CI 1.09, 2.76). Initial SEM analysis revealed no conclusive effects of stigma on drug dependence (B = − .02, 95% CI − .31 to .27, *P *= .89) or social support on anxiety (B = − .10, 95% CI − .24 to .04, *P *= .17), so these paths were trimmed in the final model. Due to the high correlation between anxiety and depression (*r *= .94) indicating potential multicollinearity, we performed sensitivity analysis in which we compared models with both variables or only one or the other. In all models including depression, there was no clear association between depression on ART adherence, either in bivariate analysis (see Table 1) or in the SEM model containing both anxiety and depression (B = .16, 95% CI .58 to − .26, *P *= .46). Given these results we removed depression from the final model. Among the covariates in the model, only self-identifying as African American/Black was associated with lower ART adherence, a finding consistent with prior studies [65–67].

**Table 1:** Correlation matrix and descriptive statistics for SEM variables (N = 317)

	2	3	4	5	6	7	8	9	10	Mean [SD] or proportion
1. VL < 50	.392^c^	− .140^b^	− .091^a^	− .098^a^	− .051^a^	.039	− .032	− .058	.029	55.2%
2. ART Adherence		− .090	− .233^b^	− .250^c^	− .130^a^	.101^a^	− .082	− .148^a^	.075	55.7%^d^
3. Depression			.937^c^	.157^a^	.515^c^	− .209^c^	.419^c^	− .062^a^	− .244^a^	1.77 [.63]
4. Anxiety				.117	.408^c^	− .164^c^	.202^c^	− .059^a^	− .177	1.95 [.70]
5. Drug Dep					.048	− .181^b^	.038^a^	.060^a^	− .133	.52 [.83]
6. Stigma						− .347^c^	.431^c^	.015	− .158	2.20 [.88]
7. Social Support							− .183^b^	.021	.189	2.44 [1.01]
8. HIV Symptoms								− .053	.023	4.06 [.16]
9. AA Race									.057	64.5%
10. SES										2.31 [1.00]

*VL* viral load count, *SD* standard deviation, *SES* socioeconomic status

^a^< .05, ^b^< .01, ^c^< .001, ^d^Among 298 participants on ART

**Fig. 1:**
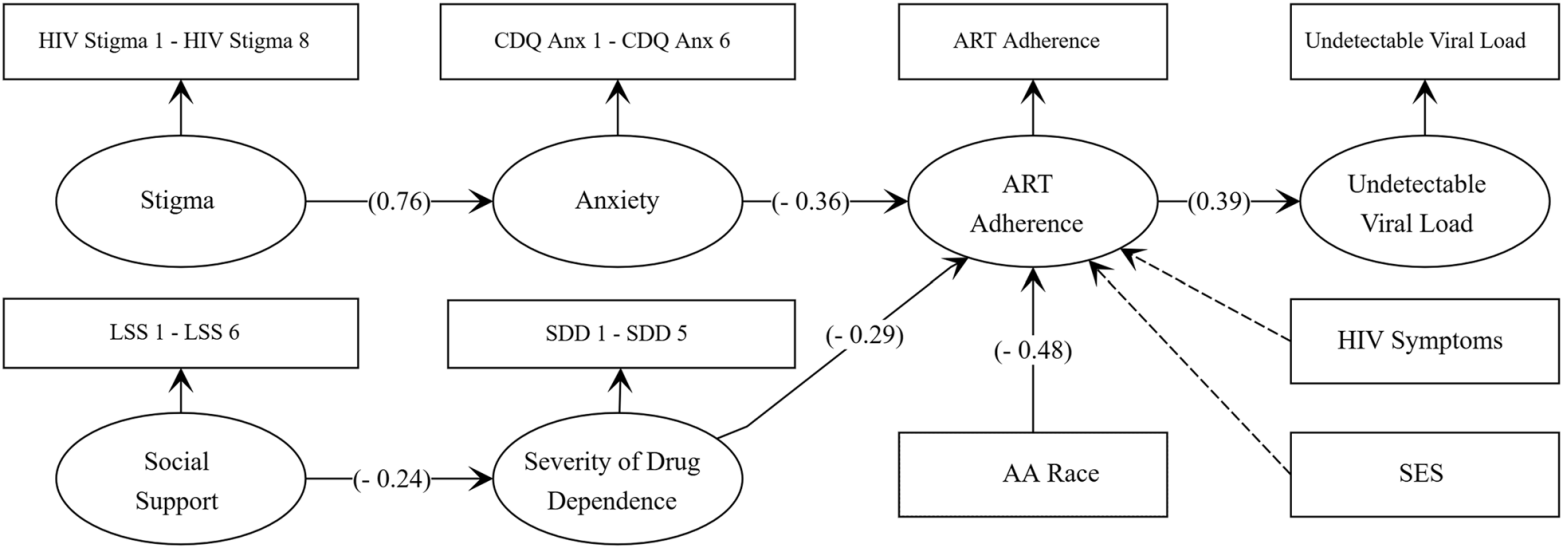
SEM path diagram showing unstandardized path coefficients. Dotted paths indicate inconclusive results (i.e., 95% CIs include nil value)

The final SEM model (Fig. 1, Table 2) showed that anxiety mediated the relationship between stigma and medication adherence, such that, on average, a 1 unit increase on the stigma scale was associated, through heightened anxiety, with a 27% lower probability of optimal adherence. Likewise, severity of drug dependence mediated the relationship between social support and ART adherence, such that, on average, a 1 unit increase on the social support scale, and associated decrease in drug dependence, resulted in a 7% higher probability of optimal adherence. In the final model, the only covariate conclusively associate with ART adherence was race/ethnicity: African American/Black racial identity was associated with a 48% lower probability of optimal adherence (Table 2). The model fit indices exhibited inconsistent results (Table 2): Chi-Square and Comparative Fit Index (CFI) indicated a moderate to poor model fit to the data, whereas the Weighted Standardized Root Mean Square Residual (WSRMR) and Root Mean Square Error of Approximation (RMSEA) indicated a good to very good model fit [68].

**Table 2:** SEM with unstandardized regression coefficients (95% CIs) for direct and indirect effects of predictor variables on ART adherence (DV); model fit indices are also shown (N = 317)

Exogenous variable (EV)	Mediator	EV on mediator^a^	Mediator on ART adherence (DV)^b^	Indirect effect EV on ART adherence^b^
Stigma	Anxiety	.764 [.401, 1.126]	− .356 [− .606, − .106]	− .272 [− .473, − .071]
Social support	Drug dependence	− .236 [− .414, − .059]	− .294 [− .482, − .107]	.070 [.003, .136]

Covariates	Estimate^b^		
Black/AA	− .484 [− .886, − .081]		
SES	1.318 [− 2.781, 5.416]		
HIV symptoms	− .008 [− .069, .054]		

Model fit	Estimate
Chi-Square	614.23; df = 476; P < .0001
RMSEA	.030 [90% CI .023, .037]
CFI	.868
Weighted SRMR	.954

^a^Linear regression estimates

^b^Probit regression estimates

**Fig. 2:**
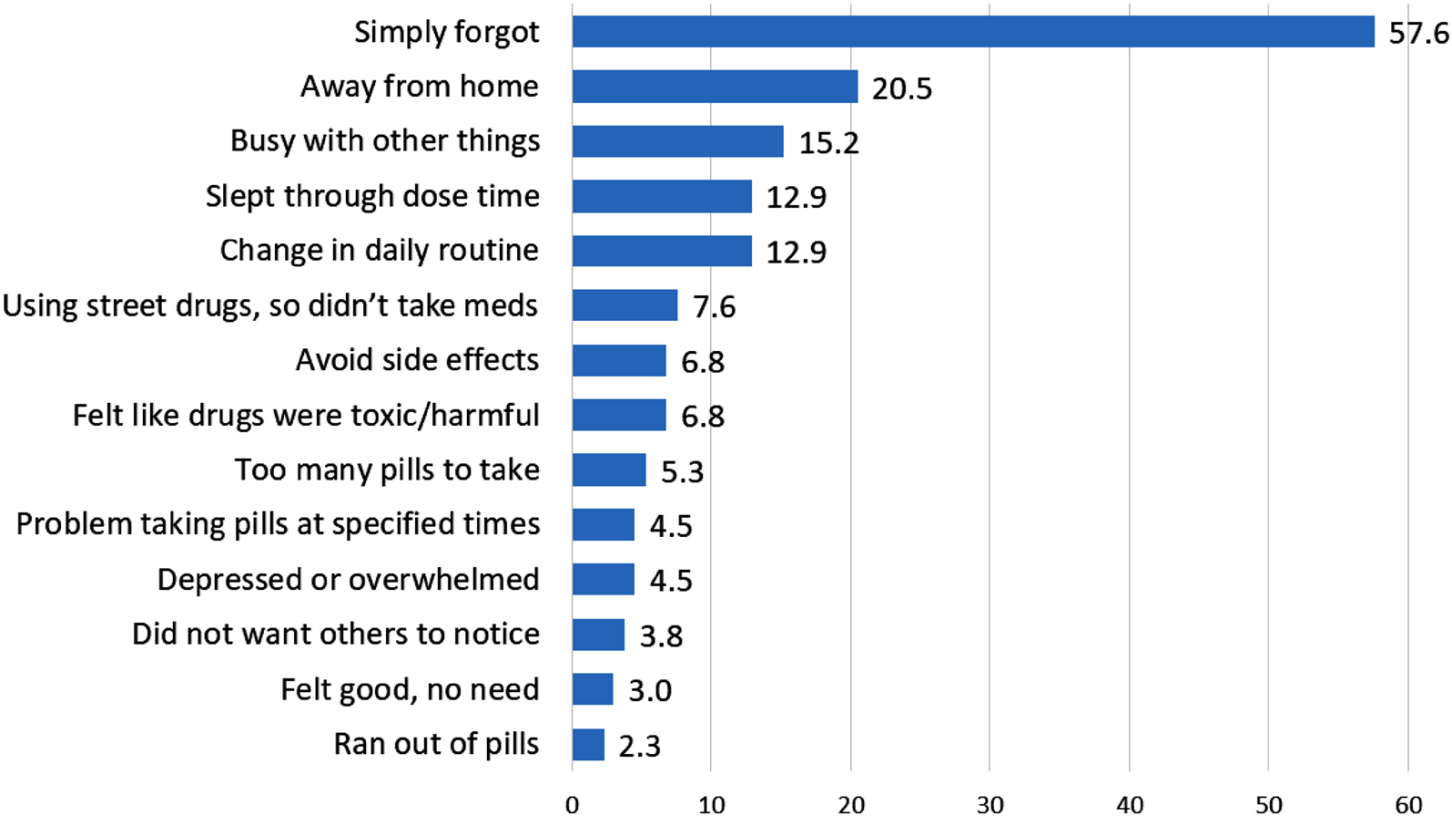
Frequency of men’s self-reported reasons for missed ART doses (n = 132)

Among participants who reported suboptimal adherence in the past month (n = 132), the most common reasons for missed doses were: “simply forgot” (57.6%), “away from home” (20.5%), “busy with other things” (15.2%), “change in daily routine” (12.9%) and “slept through dose time” (12.9%) (Fig. 2).

## Discussion

ART adherence is essential for viral control and concomitant good health and prevention of secondary HIV transmission among PLWHA; however, the factors associated with optimal ART adherence among HIV-positive adult heterosexual men remain understudied. Just over half of the men in this study reported optimal ART adherence and undetectable viral load. These findings suggest much room for improvement in these two important stages of the HIV care continuum. The results are consistent with surveillance data from the Center for Disease Control and Prevention (CDC), which indicate 55.0% viral suppression (< 200 copies/ml) among HIV-positive males assigned to a heterosexual contact risk category [69]. However, given that nearly all of the participants in the current study were receiving HIV care, a more comparable group is the CDC subsample of heterosexual men who received at least 1 VL test in past year, among whom 84% achieved viral suppression. The higher threshold of < 200 copies/mL employed by the CDC compared to our cutoff of ≤ 50 copies/mL only accounts for a few percentage points difference (data not shown). In addition, research has shown that older adults with HIV typically have higher rates of ART adherence [70]. Thus, our results indicate a much lower than expected prevalence of viral suppression among primarily older Black and Hispanic heterosexual men.

Our initial SEM mediation model did not support several components of our original hypotheses. Specifically, we found no conclusive evidence of a clear association between depression and ART adherence. A systematic review of 52 studies on the impact of depression on ART adherence found inconsistent results, with 30 studies indicating a direct negative effect of depression on adherence and 22 studies finding no conclusive effect [71]. More recent studies have also reported that depression was not directly associated with ART adherence [46, 72]. Our initial model also revealed our data was not compatible with an association between HIV-related stigma and severity of drug dependence or between social support and anxiety.

After trimming these paths, we assessed a final model involving dual indirect pathways in which anxiety and drug dependence mediated the effects of stigma and social support (respectively) on ART adherence, adjusting for race, HIV symptoms, and socioeconomic status. Higher levels of anxiety and greater severity of drug dependence were both found to have a direct negative impact on ART adherence. Further, anxiety was found to have an indirect effect of HIV-related stigma on adherence, whereas the detrimental effect of severity of drug dependence on ART adherence was attenuated by higher social support.

The negative impact of drug use on optimal ART adherence observed in this study is consistent with prior research indicating that substance use disorders are one of the strongest and most consistent predictors of poor ART adherence across diverse populations [5, 44, 67, 73–82]. Only a few studies have examined the influence of anxiety disorders on ART adherence [83]. Similar to our findings, Blake Helms et al. [84] found that attachment-related anxiety was associated with poorer ART adherence and mediated the effect of stigma on adherence.

The literature pertaining to the effects of HIV-related stigma on ART adherence has not provided consistent findings: some studies demonstrated a direct negative association whereas other studies have reported inconclusive results [76, 85]. Turin et al. [86] proposed a comprehensive conceptual framework specifying the mechanisms by which HIV-related stigma impacts HIV care and related health outcomes. In their model, four types of HIV-related stigma (enacted, community, anticipated, and internalized) affect HIV-outcomes, including ART adherence, only indirectly through interpersonal, psychological, mental health, and stress-related factors. Prior studies that support this framework have reported substantial indirect effects on the relationship between HIV-related stigma and ART adherence, including depression [87–89], overall mental health [90], adherence motivation [91] and self-efficacy [92, 93]. Several of these analyses found no direct effect of stigma on adherence in the mediation model (e.g., [88, 90]). Also consistent with the Turin model, our cross-sectional study provides evidence supporting further research into the hypothesis that HIV-related stigma might increase anxiety, which in turn leads to poorer ART adherence among HIV-positive adult heterosexual men.

Several studies have linked higher levels of social support to improved ART adherence [46, 47, 94–96]. Consistent with our findings, however, Berghoff et al. [97] found no conclusive direct effect of social support on ART adherence among a sample of primarily male, heterosexual, Black/African American HIV + patients recruited from community-based clinics. Moreover, several studies have found that social support had an indirect effect on ART adherence through various mediators, including negative affect [98], motivation [99], self-efficacy [98, 100], positive states of mind [101], elements of positive coping [102], and anxiety and depression [48]. Our analysis provides support for further research to test the hypothesis that greater social support may play a substantial role in attenuating severity of drug dependence among HIV-positive heterosexual men, which will foster improvements in ART adherence.

Consistent with prior evidence [103], the most common reasons reported by study participants for missed ART doses involved cognitive or lifestyle factors (e.g., simply forgot, away from home, slept too late, change in daily routine). These responses remind us that there are even more proximal causes of suboptimal ART adherence that are intermediate between syndemic factors and adherence behavior, and that these more distal predictors, such as stigma, social support, anxiety and substance use, are generally not recognized by HIV-positive men as contributors to ART adherence. This has implications for the development of adherence interventions, which need to address patient awareness of and inter-relationships among factors affecting adherence at multiple levels; and also for further research to elucidate the connections among more distal structural and psychosocial factors and proximal causes involving daily routine and activities, decision-making, and cognition [43].

Our study has a number of limitations. Due to the cross-sectional design of the study, our results should be viewed as hypothesis-generating rather than hypothesis-testing, as no robust causal inferences can be made. As with other studies examining ART adherence, our data will likely contain measurement error. Moreover, our models omitted potentially important health system predictors such as healthcare access and trust, as well as structural predictors such as lack of stable housing and food insecurity [104, 105], although we did adjust for socioeconomic status. In addition, our models did not specify any moderated mediation effects among the syndemic factors. Few syndemic studies examining the effects of psychosocial factors on HIV-related outcomes have explored such interactions [58]. Future research should explore the synergistic influence of these conditions on ART adherence. Relatedly, we lacked sufficient power to perform subgroup analyses to examine racial/ethnic differences in model outcomes. Finally, even with our two-stage sampling method, sampling bias may have occurred due to voluntary participation. In addition, despite our previously described efforts to recruit men ages 18–35, all participants were 33 years of age or older; thus, it is not possible to generalize our results to younger men. Such men may experience different levels of stigma, social support, or psychosocial harms compared to older men, which in turn may impact their ART adherence [106, 107].

## Conclusions

Severity of drug dependence and heightened anxiety were found to be the most important predictors of lower ART adherence among adult HIV-positive heterosexual men. These factors are known to impair psychosocial and cognitive functioning critical to optimal ART adherence. However, evidence indicates that with adequate drug and mental health treatment, HIV-positive persons with substance use and mental health disorders can achieve higher levels of ART adherence [108]. Our findings, if replicated with more robust study designs, suggest that HIV treatment programs for HIV-positive heterosexual men should include screening for co-occurring syndemic conditions, and as needed, interventions should include drug and mental health treatment approaches that incorporate social support and counseling to address HIV-related stigma [40].

## Data Availability

The datasets used and/or analyzed during the current study are available from the corresponding author on reasonable request.
